# Minimal perception: enabling autonomy in resource-constrained robots

**DOI:** 10.3389/frobt.2024.1431826

**Published:** 2024-09-18

**Authors:** Chahat Deep Singh, Botao He, Cornelia Fermüller, Christopher Metzler, Yiannis Aloimonos

**Affiliations:** ^1^ Perception and Robotics Group, Department of Computer Science, University of Maryland, College Park, MD, United States; ^2^ Perception, Robotics, AI and Sensing (PRAISe) Lab, Department of Mechanical Engineering, University of Colorado, Boulder, CO, United States; ^3^ UMD Intelligent Sensing Laboratory, Department of Computer Science, University of Maryland, College Park, MD, United States

**Keywords:** frugal AI, minimal AI, resource-constrained, autonomy, navigation, depth estimation, optical flow

## Abstract

The rapidly increasing capabilities of autonomous mobile robots promise to make them ubiquitous in the coming decade. These robots will continue to enhance efficiency and safety in novel applications such as disaster management, environmental monitoring, bridge inspection, and agricultural inspection. To operate autonomously without constant human intervention, even in remote or hazardous areas, robots must sense, process, and interpret environmental data using only onboard sensing and computation. This capability is made possible by advancements in perception algorithms, allowing these robots to rely primarily on their perception capabilities for navigation tasks. However, tiny robot autonomy is hindered mainly by sensors, memory, and computing due to size, area, weight, and power constraints. The bottleneck in these robots lies in the real-time perception in resource-constrained robots. To enable autonomy in robots of sizes that are less than 100 mm in body length, we draw inspiration from tiny organisms such as insects and hummingbirds, known for their sophisticated perception, navigation, and survival abilities despite their minimal sensor and neural system. This work aims to provide insights into designing a compact and efficient minimal perception framework for tiny autonomous robots from higher cognitive to lower sensor levels.

## 1 Introduction

Nature has spent 3.8 billion years on research and development in genetic evolution. Over the generations, living beings have evolved based on their daily activities, habitats, and surrounding environments. This natural evolution has been purposive (or *parsimonious*) rather than generic, primarily driven by perceptual behaviors tailored to specific needs and conditions. One may say that the path to evolution is *frugal*. Over millennia, these systems have become highly efficient at solving specific tasks. Such parsimonious systems, or living beings, provide a blueprint for developing the next-generation of robots. The essence of parsimony lies in utilizing minimal information or sensing modalities to achieve goals efficiently. In contrast, the field of robotics and Artificial Intelligence (AI) has been in development for just 50 years, with much of this time spent on developing independent and generic modules. Inspired by nature, robot autonomy frameworks that rely solely on onboard sensing and computation can be built, exploring new possibilities at robot scales that were never thought possible before. The path to robot autonomy lies at the intersection of AI, computer vision, robotics and sensing–leading to the tiny palm-sized parsimonious robots that excel in resource-limited settings.

Robot autonomy within a resource-constrained environment is a complex and challenging task that requires intricate strategies for optimal functionality. The core concept involves creating robotic systems that can independently carry out tasks with limited computational power, energy availability, or sensor capabilities. This becomes especially crucial in contexts like aerial robotics, deep-sea exploration, or space missions, where managing resources is essential. Algorithms such as reinforcement learning or genetic algorithms optimize resource use dynamically. These algorithms are tasked with balancing resource consumption against the quality of task execution to enhance efficiency. Additionally, sensor fusion is pivotal for compensating for limited sensor capabilities, combining data from various sources to enrich comprehension and precision. Both software and hardware are intricately co-designed to exploit specific hardware characteristics for improved resource utilization. Furthermore, autonomy in resource-constrained robots involves a comprehensive integration of planning, learning, perception, and decision-making, ensuring effective operation under challenging conditions.

**FIGURE 1:**
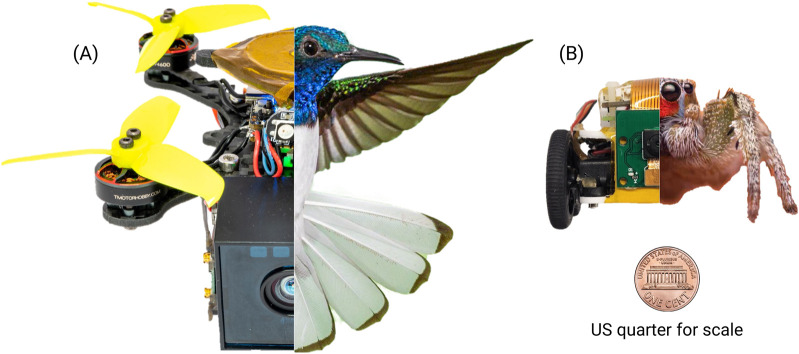
Tiny autonomous robots that utilize our Minimal Perception framework. **(A)** Autonomous drone of ∼ 120 mm in diagonal length and compared with hummingbird. **(B)** Autonomous car of about ∼ 70 mm in length and compared with a wolf spider.

From the perception perspective, robot autonomy in resource-constrained environments poses unique challenges and requires creative solutions. The perception system of a robot, which might include cameras, lidar, sonar, and other sensors, acts as its ‘eyes’. However, these sensors’ capabilities might be limited in environments where resources are constrained. To overcome this, techniques such as sensor fusion are essential. Sensor fusion integrates data from various sensor types to enhance the overall understanding of the environment and reduce perceptual uncertainty. Additionally, deep learning and computer vision methods are employed to identify relevant features from the sensory data and recognize objects and patterns. However, these techniques need to be efficiently executed due to constrained computational resources. Energy considerations are also critical, as continuous data collection and processing can consume significant power. Strategies like low-power modes and selective perception—where only pertinent data are processed—are vital. Thus, in resource-constrained robot autonomy, the perception system must carefully balance the depth and detail of environmental understanding, computational demands, and energy efficiency to maintain reliable functionality. Figure 1 illustrates tiny robots that utilize our framework. It shows the tiny scale of robots that cannot carry existing conventional sensors and computers.

Although computationally intensive perception algorithms can be offloaded to cloud computers or companion computers via networking, this raises an essential question: ‘Why do we need onboard autonomy?’. Autonomous systems that depend on wirelessly connected companion computers nearby or cloud computing face challenges in wild deployments. Such systems are vulnerable in GPS-denied environments and are often subject to latency issues. Onboard robot autonomy enhances system security, reducing vulnerability to hacking and other security threats and increasing robustness. While there are capable autonomous robots with substantial onboard computing that are relatively large (over 300 mm) for both aerial and ground applications, another question arises: ‘Why do we need small robots?’ Small robots are safe and agile and can be deployed in swarms, making them highly scalable and cost-effective to produce. Additionally, these autonomous swarms allow robots to navigate and inspect confined or hazardous areas that are time-sensitive, such as thermonuclear power plants. It is well understood that robot autonomy is significantly influenced by factors like memory speed and size, sensor type and quality, and required power. These factors directly impact the robot’s size, area, and weight.

Despite significant differences in size, area, weight, power, computation, and sensor capabilities, creatures like bees and birds can perform comparable tasks. However, their sensing and computation can differ based on their body structure and environment. Biomimetics, or biologically inspired engineering, provides essential insights for designing and creating robots by studying natural systems such as animals, birds, insects, and plants. For instance, the visual processing capabilities of a dragonfly, which can detect movement and measure depth with remarkable accuracy, inspire the development of machine vision algorithms for robotics. Similarly, the sonar system of bats, essential for navigation and hunting in complete darkness, serves as a blueprint for crafting robust echo-based sensing mechanisms, particularly beneficial for robots functioning in low-visibility conditions. Birds, adept at adjusting their flight dynamics in response to wind variations, provide valuable insights into developing adaptive perception and control systems for aerial drones. The tactile sensitivity of rodents’ whiskers also offers guidance for designing touch-based perception systems for robots navigating dark or cluttered environments. Moreover, swarm robotics frequently takes cues from ants’ and bees’ communication and coordination strategies, enabling them to perceive their environment and execute complex tasks efficiently and collectively. Therefore, perception research in biomimetics focuses on interpreting and leveraging nature’s sophisticated sensory systems to advance robotic perception and interactive capabilities.

**FIGURE 2:**
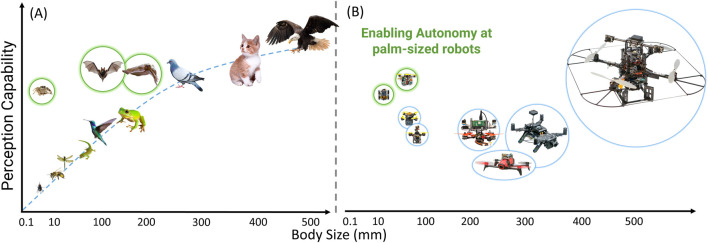
A qualitative comparison of **(A)** living beings and **(B)** robots regarding perceptual capabilities for their scaled body length. Observe the anomaly in **(A)** presented in green bubbles: jumping spider, bat, and cuttlefish. *Note that cat and eagle sizes are not to scale.*

Thus, nature inspiration is a blueprint for developing the next-generation of tiny robots with onboard autonomy capabilities. It is key to significantly scaling down current autonomous systems while maintaining or enhancing their capabilities. Refer to Figure 2, which illustrates the perception capabilities of various organisms relative to their body lengths. It is important to recognize that, generally, perception capabilities increase with body size, meaning larger organisms have more mature perception systems. However, there are notable exceptions, such as jumping spiders, cuttlefish, and certain species of frogs. For instance, jumping spiders possess a low-resolution vision system that effectively processes fast-moving objects, allowing them to respond and capture prey swiftly. Meanwhile, cuttlefish and some frogs have evolved their visual systems by altering the shape of their apertures, such as the ‘W’-shaped aperture in cuttlefish and vertical or horizontal openings in some frogs. Additionally, the blue and green bubbles in Figure 2 represent real-world tiny robots with onboard autonomy. Previously, robots as small as 120 mm could perform tasks like navigation, obstacle avoidance, and maneuvering through gaps of unknown shapes (Jagannatha Sanket, 2021), as indicated by the blue bubble. This work progresses, enabling even smaller robots–as small as a credit card (less than 3 inches in length) – to achieve enhanced autonomy, as shown in the green bubble.

To achieve scalability, sustainability, and distributability in these robots, small palm-sized robots must be built capable of performing parsimonious tasks. In this work, we introduce a minimal perception framework that is at the heart of robot autonomy for palm-sized robots. This principle of simplicity guiding complexity should also inspire our approach to robotics—where we aim to evolve complex functionalities out of simple designs, focusing on efficiency rather than excess. One of the profound influences on this idea comes from Noam Chomsky’s Minimalist Program in Linguistics. Chomsky, a distinguished linguist, cognitive scientist, and philosopher, introduced this program as a fundamental reevaluation of syntactic theory, suggesting that nature, including human language, functions as straightforwardly and efficiently as possible. At the heart of Chomsky’s theory is that sentences are constructed from a basic set of lexical items through binary merges. This minimizes computational complexity by consistently using the same operations to structure sentences. This allows for an endless variety of expressions from a limited number of elements. Another key aspect of the Minimalist Program is the principle of ‘economy,’ which posits that linguistic expressions adhere to a principle of using the least resources necessary, reflecting the minimalist credo that ‘less is more.’

**FIGURE 3:**
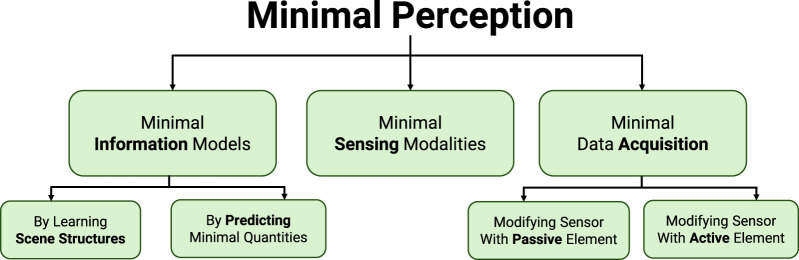
Minimal perception framework.

We introduce a *Minimal Perception* framework (see Figure 3) that takes inspiration from nature and addresses the frugality or minimalism at all levels–from higher cognitive to lower sensory levels. This sits at the heart of autonomy frameworks for resource-constrained robots. The purpose of this work is to introduce the methodology of minimal perception to the robotics community. To achieve it in the confines of this paper, we had to rely on a number of results from our previous and current research along with novel concepts. Specifically, we utilize our results from Ajna (Sanket et al., 2023), TinyDepth (Singh, 2023), CodedVO (Shah et al., 2024b) and AMI-EV (He et al., 2024) to show a taxonomy of perception-action questions. This taxonomy constitutes the minimal perception framework.

### 1.1 Related works

Active vision (Aloimonos et al., 1988; Bajcsy et al., 2018; Bohg et al., 2017; Jagannatha Sanket, 2021) is a dynamic approach in computer vision and robotics where a system controls where it focuses its attention instead of passively scanning the entire scene. This often involves physically moving the sensor or altering the environment. It mimics the way humans and animals actively look around to collect information. GapFlyt (Sanket et al., 2018) explores these behaviors and computes optical flow from various perspectives to estimate ordinal depth. However, one major drawback of this approach is its lack of mathematical guarantees for navigating through gaps since it cannot compute metric information.

The use of neuromorphic sensors is another minimal source of information that has become ubiquitous in robotics today. (Falanga et al., 2020). addresses the problem of dodging spherical objects on drones. EVDodgeNet (Sanket et al., 2020a) furthers this research with avoidance and pursuit tasks for unknown dynamic obstacles. (Xu et al., 2023). introduces a bio-inspired architecture for event camera drone avoidance.

Sparse sensing has also been extensively studied to enable autonomy on tiny robots (Duisterhof et al., 2021). introduces learning on tiny robots (Ostovar, 2022). introduces indoor collision avoidance with sparse time-of-flight depth sensors. (Müller et al., 2023). develops robust and efficient depth-based avoidance systems for miniaturized UAVs (Friess et al., 2024). demonstrates an onboard SLAM method for distributed mapping on a swarm of nano drones (Müller et al., 2024). illustrates using ultrasound sensors for low-power robot autonomy.

Researchers have leveraged camera models to estimate metric depth from single images in computational imaging by altering the camera apertures (Gopinathan et al., 2003). introduces custom coded apertures to existing cameras for efficient motion tracking (Asif et al., 2016). introduces a thin and lensless camera using a coded aperture that makes the camera systems smaller. The use of passive elements on the aperture plane is a well-explored concept, particularly in applications such as light-field imaging (Veeraraghavan et al., 2007) and depth estimation (Levin et al., 2007; Zhou et al., 2011; Takeda et al., 2013). It is understood that the depth-dependent defocus, known as ‘*bokeh*’, or the point spread function (PSF), is influenced by the amplitude and phase of the aperture employed. The two most commonly used coded apertures are the amplitude mask (Levin et al., 2007) and the phase mask (Wu et al., 2019).

## 2 Materials and methods

The underlying principle of minimal perception is to extract only the essential information to perform a given task while optimizing resource utilization in autonomous systems. The principles that govern the minimal perception framework are as follows:

• Selective Information: Minimal perception involves the selective extraction of useful information from the environment. The fundamental idea is to focus and prioritize relevant data that is useful for a task $T_{i}$ while disregarding non-essential and/or redundant data. Techniques such as salient feature selection/analysis (Simonyan et al., 2014), attention mechanism (Niu et al., 2021) and passive computing (Singh, 2023) hold the key to minimizing the computational load especially when dealing with large inputs.
• Minimum Prior Knowledge: *‘What is the information* I *required to solve* N *set of tasks* $T_{N}$ *in a given amount of time?’* This question explores the feasibility of accomplishing tasks with the least amount of prior knowledge. Strategies of Active and Interactive Perception (Bohg et al., 2017; Kragic et al., 2018) are crucial in addressing these tasks when minimal prior knowledge is available.
• Adaptive Sensing: The utilization of adaptive sensing strategies to tailor data collection to the specific context and task requirements is an essential element for a minimal perception framework. These adaptive sensing techniques modify sensor parameters dynamically, including sensing modalities and modifications to aperture shapes, to efficiently gather the required information. By adapting the sensing process to current conditions, minimal perception minimizes resource use and enhances the efficiency of data acquisition.
• Attention Mechanism: Attention mechanisms play a crucial role in minimal perception by directing computational resources on stimuli. Inspired by nature’s visual attention, these mechanisms distribute processing power and sensory attention to the most pertinent aspects of the input data. By selectively concentrating on essential features or areas, minimal perception enhances computational efficiency and supports real-time responsiveness in systems with limited resources. Section 2.2 demonstrates this principle by illustrating the robot’s ability to estimate dense depth across all spatial directions. However, it strategically allocates its computational resources primarily to the direction that presents the highest potential risk, thereby ensuring effective obstacle avoidance and navigation during tasks.
• Hardware-Software Optimization: To quote a famous researcher, Alan Kay, “People who are serious about software should make their own hardware.” Hardware-software co-design is crucial for enhancing the functionality and performance of mobile robots. This approach involves the integrated optimization of hardware components and software algorithms tailored to meet the specific needs of mobile robotic applications. The co-design process balances computational efficiency, power consumption, real-time responsiveness, and physical limitations. This balance enables mobile robots to effectively navigate environments, execute complex tasks, interact with humans, and adjust to dynamic conditions.

The notion of minimal perception can be conceptualized at different levels–from higher cognitive to lower sensor levels. We classify minimal perception into three different hierarchical levels (Figure 3) – (a) Minimal Information Models, (b) Minimal Sensing Modality, and (c) Minimal Data Acquisition. Table 1 illustrates the difference between minimal perception and traditional approaches for every aforementioned principle. Note that these principles are valid at each of the three hierarchal levels.

**TABLE 1:** Minimal perception *versus* traditional methods (maximal perception).

Principle	Minimal perception	Maximal perception
Information Selection	Extracts only essential data from sparse or minimal sensing	Extracts comprehensive data from dense sensor arrays, including LiDAR and multiple cameras
Prior Knowledge	Low Prior Knowledge. Gathers necessary and latent information through environmental interaction	Higher Prior Knowledge. Utilizes extensive pre-built maps and databases for navigation and task execution
Sensing Adaptiveness	Utilizes lightweight sensors with dynamic parameter adjustments based on task requirements	Uses high-resolution, fixed-parameter sensors for detailed environment mapping
Attention Mechanism	Focuses computational resources on critical tasks through attention-based processing	Applies brute-force processing power to manage all sensor data simultaneously
Hardware Software Optimization	Integrates simple hardware with efficient software algorithms to maximize performance per watt	Uses powerful processors and GPUs to handle complex algorithms and large volumes of data

### 2.1 Minimal information models

The classical theory of visual perception, which relies on single images tailored for static scenes, has been highly successful. However, it falls short in dynamic real-world environments, posing limitations on robot autonomy. By incorporating motion or *Temporal Information* (TI) alongside sensor characteristics, we’ve unlocked previously untapped potentials in perception. Utilizing TI allows us to address common robotics challenges, such as navigation and segmentation, without needing depth or range sensors. To achieve these tasks, the robot must understand the environment’s geometry and the physics of its movements rather than solely focusing on scene characteristics. Let us look at an example that depicts the importance of observing the environment from multiple views.

**FIGURE 4:**
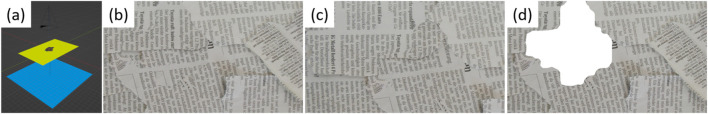
Segmentation of the gap with similar texture on the foreground and background elements. **(A)** 3D model of the scene. **(B,C)** Snapshots of consequent timestamps. **(D)** Segmentation result.

Figure 4 depicts a scene with a foreground and a background. In Figure 4A, the side view setup is shown, with the yellow region representing the foreground containing a gap or hole and the blue region representing the background. Notably, both foreground and background elements have identical textures. Observing the scene from a single view, as depicted in Figure 4B, it is impossible to determine the precise location of the gap. However, by combining Figures 4B, C, optical flow calculation becomes feasible, enabling the estimation of ordinal depth. Consequently, the gap in the image can be identified, as demonstrated in Figure 4D.

Active vision strategies have the potential to greatly augment the functionality of automated systems in real-world scenarios, including autonomous vehicles, industrial robots, and surveillance systems. For instance, an autonomous vehicle outfitted with an active vision system can dynamically adjust its sensors to focus on specific points of interest, such as pedestrians, road signs, or other vehicles. Likewise, in surveillance applications, an active vision system can prioritize monitoring unusual movements or behaviors, thereby enhancing the overall effectiveness and efficiency of the system. Consequently, active vision is pivotal in advanced AI systems, enabling them to engage more proficiently with their surroundings.

GapFlyt (Sanket et al., 2018) investigates these behaviors and introduces ${TS}^{2}$ P, where optical flow (Ilg et al., 2017) from various viewpoints is obtained and stacked to estimate ordinal depth. However, a notable limitation of this technique is the lack of mathematical assurances for successful gap traversal. The drone cannot estimate the metric depth of the gap or determine whether it has successfully traversed through it.

Temporal information (TI) also introduces uncertainty into network predictions, a feature currently exploited by roboticists and computer vision scientists to enhance accuracy. However, these uncertainties harbor hidden insights with considerable potential for addressing various robotics challenges. Aleatoric uncertainty, in particular, characterizes biases inherent in sensor data collection, such as cameras’ limited perception of obstructed objects. To illustrate the potential of these additional cues, aleatoric uncertainty prediction was exclusively applied to TI, specifically optical flow, for a range of robotics applications. The primary advantage of relying solely on uncertainty rather than traditional predictions is a significant reduction in computational costs, often by a factor of 10–100. Works like Ajna (Sanket et al., 2023) exemplify real-time robotics tasks utilizing uncertainty, including navigation, static and dynamic obstacle avoidance, traversal of unknown gaps, and segmentation. Substantial uncertainties were observed in challenging areas of optical flow, such as occlusions and motion blur, effectively aiding obstacle detection within the scene. Moreover, a promising class of sensors known as neuromorphic sensors or event cameras, capable of extracting TI at the sensor level, holds the potential for further enhancing robot efficiency.

While uncertainties are valuable for integrating multiple measurements, we believe their potential in robotics remains largely untapped. This is primarily because uncertainties offer contextual insights beyond their combined capabilities. Before exploring specific examples, let’s introduce two common types of uncertainties: Aleatoric, also known as observational data uncertainty, and Epistemic, which relates to model uncertainty. Aleatoric uncertainty reflects the sensor’s inherent bias in data collection, while epistemic uncertainty stems from the scenarios used to train the model. For instance, aleatoric uncertainty would be significant in transparent or dark regions when using RGB-D data. In contrast, when applied to outdoor data, a network trained indoors would exhibit high epistemic uncertainty. Estimating epistemic uncertainty demands variational inference and multiple neural network runs, often impractical for real-time applications without employing multiple accelerators. Conversely, aleatoric uncertainty suits real-time use, requiring minimal parameter increases and a single network pass for prediction. This study focuses on estimating heteroscedastic aleatoric uncertainty, offering specific observational uncertainty insights for input data.

We address the following question–*How can we estimate heteroscedastic aleatoric uncertainty in neural networks? What informational cues does it provide for various robotic tasks?* This work presents a novel generalized approach for heteroscedastic aleatoric uncertainty in neural networks.

Consider an input x to a neural network N with weights W . The estimated output of the network N is represented by $\tilde{y}$ (Equation 1), while the actual prediction is denoted by $\hat{y}$ .

$$\tilde{y}=N\left(x|W\right)$$ (1)

We optimize the following problem by learning the weights W using Equation 2:

$${\mathrm{arg min}}_{W,\Psi }f\left(\hat{y},\tilde{y}\right)\;\text{s.t.}\;\Psi =k\left(f\left(\hat{y},\tilde{y}\right),x\right)$$ (2)

Here, the symbol f represents a distance metric between the predicted value $\tilde{y}$ and the ground truth value $\hat{y}$ . The symbol Ψ corresponds to a monotone function k that depends on the heteroscedastic aleatoric uncertainty of the underlying probability distribution $p\left(x,\tilde{y}|W\right)$ . This uncertainty is positively correlated with the expected error or risk. The correlation between two random variables X and Y is formally expressed as the Pearson correlation $\rho_{X,Y}$ , where the symbol E denotes the expectation operator.

From (38), we can say that Ψ symbolizes the predicted output’s expected error, risk, or uncertainty. A self-supervised optimization of the following function is required to calculate Ψ , hereafter referred to as “uncertainty” for clarity.w

$${\mathrm{arg min}}_{\tilde{y},\Psi }\left(\lambda g\left(\Psi \right)+f\left(\hat{y},\tilde{y}\right)h\left(\Psi \right)\right)$$ (3)

In the optimization function above, the function g signifies a monotonic relationship with uncertainty, preserving domain order and convexity. Conversely, the function h reverses the monotonicity of g , ensuring $\rho_{h,g}<0$ (where h might also depend on g ). This formulation aims to establish a two-way coupling between Ψ and $\tilde{y}$ to avoid trivial solutions and scale the values appropriately.

In the presented formulation, Ψ can represent either uncertainty (similar to covariance) or lack of confidence (risk) of any arbitrary distribution. For intricate distributions, Ψ may become a complex function of the variance ν , resulting in qualitative uncertainty rather than quantitative. However, by judiciously selecting functions f , g , h , and λ , Ψ can be transformed into a quantitative function of ν with straightforward closed-form solutions. In such instances, it is also feasible to strive to certify the robustness of neural networks within a confined training/operating data domain.

Now, we address the question – ‘What informational cues does the uncertainty hold for tiny robots?‘. The answer to this question can be found by considering the uncertainty in optical flow. Unlike GapFlyt, we are not required to compute dense optical flow for navigation. Rather, we utilize only uncertainty in optical flow to find unknown shaped gaps and dynamic obstacles. This is because the uncertainty in flow increases due to failure in optical flow, which is high in the regions with occlusions, light changes, and motion blur.

It is important to highlight that this work aims to showcase the application of uncertainty in diverse robotics tasks and how such a formulation can integrate various categories of robotics challenges. Hence, in our experiments, we refrain from incorporating additional information such as color, optical flow, or depth, except for comparative analysis in this chapter. Furthermore, we do not make any assumptions regarding the placement or type of structures used in our experiments. Our control decisions are based solely on the Ψ derived from the current image pairs without employing temporal smoothing or filtering techniques.

The results of this section are presented in Section. 3.1.

### 2.2 Minimal sensing models

Minimal sensing in robots refers to implementing a simplified sensory system that allows a robot to perceive and understand its environment with a limited number of sensors. The aim is to create a sensing setup that uses resources efficiently while providing enough information for the robot to carry out its tasks effectively. This method selects the essential sensors needed to capture critical environmental details like proximity, orientation, or object detection, tailored to the application’s specific needs. By limiting the number and complexity of sensors, robots can cut costs, reduce power use, and lessen computational demands while maintaining adequate situational awareness. The challenge is to strike the right balance between the richness of sensor input and system constraints to achieve reliable and efficient functionality in practical settings.

For autonomous robots, accurately measuring distances and understanding the geometry of a 3D scene are crucial. Robots often depend on depth maps for navigation in complex and changing environments. Traditional depth estimation techniques, whether based on single or multiple camera systems, typically require heavy computation or high-end sensors, making them impractical in settings with limited resources. An alternative is using motion cues such as parallax, seen in nature with birds like pigeons, to simplify depth calculations. Efforts have been made to reduce the computational load by decreasing resolution or utilizing predictable environmental cues. However, these methods often compromise accuracy for tasks like obstacle avoidance or do not adapt well to new or unfamiliar environments when deployed in real-world conditions.

We proposed a model that utilizes a sparse depth sensor and an RGB camera to learn and predict dense depth maps (Singh, 2023). The method proposed calculates a high-resolution metric depth map by analyzing a pair of standard color images and sparse Time-of-Flight (ToF) depth data (with an 8×8 pixel resolution) from two perspectives. This input consists of eight channels: six for RGB color information and two for sparse depth maps. Before delving into the specifics of the neural network, let’s first explore the sensor arrangement. Our setup comprises an RGB camera and a VL53L5CX sensor (or L5), firmly attached to one another.

To learn high-resolution depth maps, we first need to provide enough learning data that constitutes dense depth maps, L5 data, and RGB data. To simulate the L5 data, it is essential to understand how the VL53L5CX collects data. The L5 employs a histogram-based algorithm to compute depth. Each zone of the L5 sensor yields the mean distance (or depth) value derived from the distribution of all photons hitting that specific sensor zone. With 100 bins in each zone, the ToF L5 sensor covers a depth range of up to 400cm, with each bin representing a 4 cm interval. This design choice renders the sensor both low-power and low-bandwidth. Additionally, the L5 sensor flags any instances of insufficient samples or unstable results, ensuring that unreliable zone values are disregarded for inference.

We emulate sparse L5 data using ground truth depth maps sourced from the NYUv2 dataset (29) to train our depth maps. We resize the aligned RGB and depth images to a resolution of 320×320 pixels. To simulate L5 signals, we partition the ground truth depth image into 8×8 zones, each with a resolution of 40×40 pixels. It is important to note that we maintain the same 320×320 pixel resolution for both RGB and L5 data to expedite the learning of pixel-to-pixel correspondences between the two sensor datasets, as opposed to using 8×8 pixel L5 data for training. Within each zone, we compute the mean of the normal distribution of the depth data histogram and approximate it to the nearest binned L5 value (multiples of 4 cm), representing the L5 signal for that zone. Additionally, Gaussian noise is introduced to each L5 signal to prevent overfitting of the TinyDepth model to the L5 data. Moreover, a few L5 signals are randomly removed to simulate the unstable zones of the VL53. Notably, we refrain from normalizing the depth data, preserving it in metric units ranging from 0m to 4 m to learn the scale.

To prioritize learning the depth via geometry of the scene and prevent regression or overfitting on textures, we implement an eight-channel input strategy (320×320×8) . This input comprises six channels for RGB data and two channels for simulated L5 data, observed from two distinct viewpoints, say, $C_{1}$ and $C_{2}$ . This approach enables the integration of motion cues into our network. Assuming Lambertian surfaces within the field of view, we leverage a convolutional encoder-decoder architecture featuring skip connections, incorporating residual learning.

For our depth prediction, we adopt loss functions inspired by those commonly employed to train optical flow networks, as they have demonstrated superior generalization (Sanket et al., 2020b; Sun et al., 2018). We also add forward-backward consistency and edge-aware losses to our network for more generalizable and optimal performance in depth estimation. More network details and loss functions can be found in (Singh, 2023).

The results of this section are presented in Section 3.2.

### 2.3 Minimal data acquisition

**FIGURE 5:**
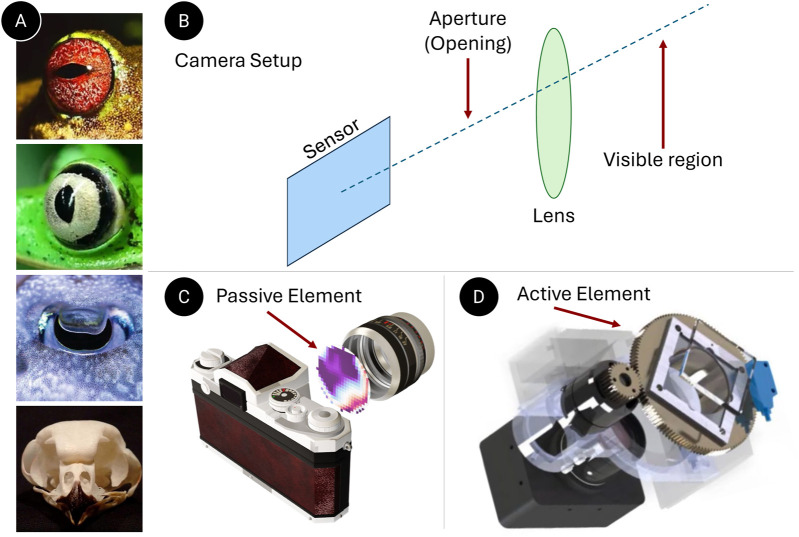
Minimal Data Acquisition: **(A)** Illustrates asymmetric perception system in nature (from top to bottom–vertical aperture in frogs, horizontal aperture in frogs, W-shaped aperture in cuttlefish and asymmetric ears in owls.) **(B)** Represents a typical camera system. **(C)** Demonstrates our data acquisition methods that use passive elements at the aperture. **(D)** This represents our method, which utilizes an active element before the lens.

While designed for various applications, autonomous agents share a common approach in their perceptual systems—the generation of a 3D scene representation (Taketomi et al., 2017). Subsequent tasks, including navigation and interaction, are planned based on this model to facilitate autonomous behavior (Marr, 1977; Thrun, 2002; Lluvia et al., 2021). This design philosophy stems largely from the perception algorithms traditionally based on the primate or human visual system, which was not initially evolved for navigational autonomy (Pentland and Addison, 1986). Applying these generalized algorithms in autonomous agents, such as drones, often results in inefficiencies due to their non-specific nature. In contrast, perceptual systems in biological entities, both visual and auditory, are adapted to their environments and have evolved to be highly efficient. For example, animals like frogs have both vertical and horizontal pupils (Figure 5A) based on their environment, habitat, and day-to-day tasks. This variation in pupil shape is not only evident across different species but also within the same species. Figure 5(A-iii) shows the W-shaped aperture of a cuttlefish. From an auditory standpoint, owls have evolved asymmetrical ears for precise sound localization. Furthermore, it is known that bats possess large ears relative to their body size to enhance their auditory capabilities. Inspired by these asymmetrical biological designs, we propose a design language of data acquisition for robot perception to process and extract latent information efficiently.

Minimal data acquisition in robot perception involves the strategic and efficient collection of sensory data essential for effective perceptual tasks in robotics. This method emphasizes the selective capture and prioritization of crucial data, enabling robots to optimize their computational resources and enhance their decision-making processes in real time. The focus is gathering only the necessary information for accurate environmental perception while excluding superfluous or irrelevant data. Active perception and sensor fusion are pivotal in reducing data acquisition. Active perception employs intelligent control strategies to direct the robot’s sensors toward specific areas of interest, thereby maximizing the relevance and utility of the data collected. Sensor fusion integrates inputs from various sensors to construct a detailed and dependable environmental model. By implementing strategies of minimal data acquisition, robots can improve their perceptual efficiency, decrease computational demands, and streamline their operations across diverse applications like navigation, object recognition, and scene interpretation.

A passive or active element can be introduced in front of the camera lens or sensor plane to modify data acquisition without additional computational work. Passive elements, like custom apertures, and active elements, such as rotating prisms, can filter data directly at the hardware level.

#### 2.3.1 Minimal acquisition via passive elements

Inspired by the evolutionary adaptations of eyes and pupils, researchers have utilized coded apertures to gather depth information using passive monocular camera systems. In this section, we propose coded apertures (see Figure 5C) – designed specifically for metric depth estimation in RGB cameras and high-speed 3D tracking of objects in event cameras.

Optical cameras capture images based on wavelength and depth-dependent blurs called point spread function (PSF) – the response of an optical system to a point source with a specific position and wavelength. A PSF can be simulated using Fourier optics theory (Goodman, 2005). The PSF h induced by the lens system of the camera with specific amplitude modulation A and phase modulation ϕ is given by Equation 4:

$$h={\left|F\left(A\exp\left(i\phi^{DF}\left(d\right)+i\phi \right)\right)\right|}^{2}$$ (4)

Where $\phi^{DF}\left(d\right)$ represents the defocus aberration due to the point source being d units away from the focal plane, and F indicates the 2D Fourier transform. Leveraging this model, coded (or blurred) images $I_{c}$ can be derived from AiF images I via a nonlinear blurring process outlined in (Ikoma et al., 2021), which can be expressed as Equation 5:

$$I_{c}=\overset{D}{\underset{d=1}{\sum }}\frac{h\;*I}{h\;*\sum_{d^{\prime }=1}^{d}O_{d^{\prime }}}\overset{D}{\underset{d^{\prime }=d+1}{\prod }}\left(1-\frac{h\;*O_{d}}{h\;*\sum_{d^{\prime }=1}^{d}O_{d^{\prime }}}\right)$$ (5)

In this equation, * denotes the 2D convolution operator and {1,…,D} denotes a set of discrete depth layers. $O_{d}$ and h are the occlusion mask and PSF at depth d , respectively.

Owing to the variation in blur patterns at different depths, metric depth can be inferred from a single coded image $I_{c}$ (Levin et al., 2007). We utilize a U-Net architecture for depth estimation, extensively employed in tasks requiring pixel-wise predictions. We introduce a depth-weighted metric loss that encourages the network to prioritize learning depth differently across various depths as detailed in Equation 6:

$$L=\frac{1}{N}\overset{N}{\underset{i=1}{\sum }}w_{{\hat{D}}_{i}}\cdot {\left(\tilde{D_{i}}-{\hat{D}}_{i}\right)}^{2};w_{{\hat{D}}_{i}}=\alpha^{-\beta {\hat{D}}_{i}}$$ (6)

Here, $w_{{\hat{D}}_{i}}$ represents depth-aware weights within our proposed L loss, which exponentially change according to the estimated depth ${\tilde{D}}_{i}$ . ${\hat{D}}_{i}$ is the ground truth depth. The parameters α and β are empirically set to 2 and 0.3, respectively.

In this work, we plug and play our predicted depth maps in existing RGB-D visual odometry methods such as ORBSLAM (Mur-Artal et al., 2015). However, for RGB frames, one could either utilize the coded image $I_{c}$ or recover AiF I image from $I_{c}$ using different refocusing techniques (Veeraraghavan et al., 2007; Elmalem et al., 2018; Saito and Saito, 2019). However, recovering I from $I_{c}$ requires additional computing and is prone to inconsistent errors. Prior computational imaging neural network-based methods (Ikoma et al., 2021; Liu et al., 2022) generate inconsistent artifacts between frames and hinder the performance of feature correspondences and tracking. Thus, we utilize coded image $I_{c}$ as a tractable alternative RGB input along with our predicted $\tilde{D}$ for RGB-D odometry methods. In-depth details of the work can be found at https://prg.cs.umd.edu/CodedVO.

Furthermore, we can achieve high-speed real 3D tracking of points in real-time using amplitude masks with event cameras. Please refer to the work in (Shah et al., 2024a) for more results.

The results of this section are presented in Section 3.3.

#### 2.3.2 Minimal acquisition via active elements

Neuromorphic vision sensors, or event cameras, have significantly advanced visual perception by offering exceptionally low reaction times, thereby paving the way for applications in high-dynamic robotics. The camera output is influenced by both motion and texture, yet they struggle to capture object edges that move parallel to the camera’s trajectory. This limitation, intrinsic to the sensor design, poses a significant challenge for algorithmic correction. In human vision, perceptual fading is countered through involuntary eye movements, notably microsaccades, which by continuously and subtly adjusting the eyes during fixation, help preserve texture stability and visibility (Rucci and Victor, 2015).

Drawing inspiration from microsaccades and previous works that enhance the perception of neuromorphic vision in an active manner (Testa et al., 2023), we have developed an event-based perception system that maintains both a low reaction time and stable texture visibility. Our approach involves the use of a rotating wedge prism positioned in front of the event camera’s aperture, which redirects light to provoke event detection (See Figure 5D). The geometric optics of the rotating prism enable algorithmic adjustments to counterbalance the added rotational movement, thereby ensuring stable texture appearance and high-quality output regardless of external motion. This integrated hardware and software system, which we have named the Artificial MIcrosaccade-enhanced EVent camera (AMI-EV), has shown superior data quality in benchmark tests, outperforming both standard and event cameras in various scenarios. Through numerous real-world tests, AMI-EV has demonstrated its capability to enhance robotic perception for a broad range of vision tasks, from basic to advanced levels.

This work addresses the key challenges in achieving accurate and stable event-driven data association, focusing on an integrated approach that combines hardware and software design. Rather than merely imitating natural mechanisms, we introduce a nature-inspired yet more sophisticated solution, the Artificial MIcrosaccade-enhanced EVent camera (AMI-EV). This system enhances event camera capabilities using a rotating wedge prism to manipulate incoming light effectively. The AMI-EV proactively triggers events in areas of high spatial frequency, such as edges, ensuring consistent texture representation and high-quality data output, even in the absence of sensor motion.

Our compensation algorithm integrates seamlessly with existing event-based perception algorithms, making the AMI-EV a ready-to-deploy solution. We demonstrate the system’s versatility and effectiveness by applying it across a spectrum of vision tasks, from basic to complex, thereby validating its potential in diverse application scenarios. For more details, please refer to (He et al., 2024).

The results of this section are presented in Section 3.4.

## 3 Results

### 3.1 Navigation via minimal information models

We evaluate our uncertainty method in (Sanket et al., 2023) on a drone for various real-time robot applications–dodging dynamic obstacles, navigating through unstructured environments, flying through unknown shaped gaps, and object segmentation via interactive perception.

This section’s experiments are conducted using a custom-designed quadrotor platform, PRGLabrador500 (Sanket et al., 2019), as shown in Figures 7A, B. This quadrotor features an X-shaped frame with a motor-to-motor span of 500 mm. It is equipped with T-Motor F80 Pro 2500 KV motors and 6042 × 3 propellers. Vision and planning algorithms run on the Jetson TX2, executing at a frequency of about 8 Hz with Python 3.6. All flight experiments occur within the Brin Family Aerial Robotics Lab at the University of Maryland, which provides a controlled environment with dimensions of 7.3×5.5×5 $m^{3}$ .

The perception pipeline processes consecutive RGB color frames with a resolution of 320×240 pixels at a frame rate of 30 Hz. These frames are fed into a modified version of the EVPropNet architecture (Sanket et al., 2021), where the number of output channels has been increased to four from the original one (Sanket et al., 2021). The modified network, named *Ajna*, comprises 2.72 million parameters and demands approximately 6.3 GFLOPs for each forward pass. The model’s size is 10.40 MB, and it performs inference in about 49 m per frame (20.4 Hz) with a batch size of one.

*Ajna* (Sanket et al., 2023) is trained using a loss function that combines self-supervised learning for uncertainty estimation and supervised learning for predicting target outcomes, as detailed in Equation 3. The network specifically aims to predict dense optical flow $\tilde{{\dot{p}}_{x}}$ and its associated dense heteroscedastic aleatoric uncertainty $\Psi_{x}$ . The initial training phase spans 400 epochs on the Flying Chairs 2 dataset (6, 16) with a learning rate of $10^{-4}$ . This is followed by an additional training period of 50 epochs on the FlyingThings3D dataset (24) using a reduced learning rate of $10^{-5}$ . A batch size of 32 is used throughout the training process. We employ this technique to quantify uncertainties in the drone images. This allows us to effectively differentiate and segment static and dynamic obstacles, facilitating successful avoidance and navigation around these objects.

**FIGURE 6:**
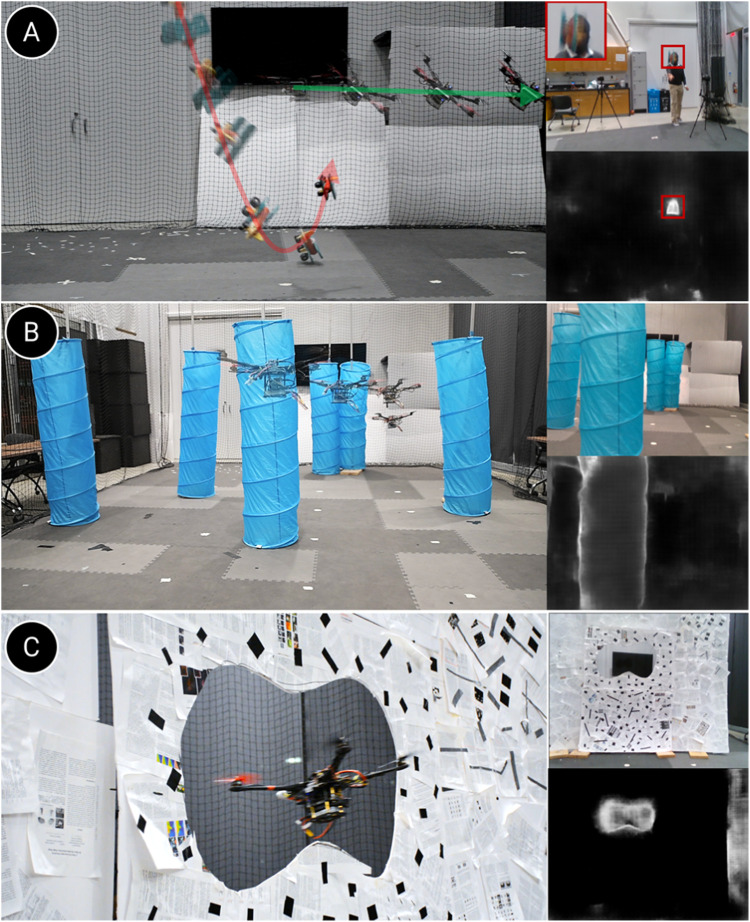
Navigation via uncertainty estimation: **(A)** Dodging dynamic obstacles, **(B)** Navigation through the indoor forest, and **(C)** Flying through unknown gaps. In each image, the left image shows a third-person view, the top right inset shows the image seen by the drone, and the bottom right inset shows the uncertainty output. Here, uncertainty ranges from 0 to 1 (black to white).

Figure 6 demonstrates the results of successfully dodging dynamic obstacles, flying through indoor spaces, and unknown-shaped gaps. For gap experiments, we achieved a detection rate (DR) accuracy of 91% across 100 trials, navigating through four different gaps of unknown shapes with a minimum clearance of just 8 cm. For dodging experiments, we achieve an overall success rate of 83.3% over 60 trails with multiple objects.

Utilizing the heteroscedastic aleatoric uncertainty Ψ of the optical flow, we identify object boundaries created by accretions and deletions, which are fundamental for conducting ‘depth-based segmentation.’ This segmentation technique is integral to addressing the four outlined tasks with a streamlined perception stack. The Ψ associated with optical flow provides additional informational cues crucial for detecting dynamic obstacles and aiding navigation, particularly through motion blur, which can render optical flow undefined. In scenarios where a dynamic obstacle moves significantly faster than the robot, the resultant motion blur leads to poorly defined optical flow estimates, resulting in high Ψ values. This effect is akin to the functionality of event cameras, where dynamic obstacles become prominently visible due to the generation of many events, a phenomenon replicated by elevated Ψ levels.

The method introduced in this study adopts a progressive baby-steps strategy by implementing a generalized uncertainty formulation. This approach allows for innovative solutions to typical robotics challenges. This methodology is expected to broaden the scope of robotic autonomy, facilitating breakthroughs in applications previously considered too challenging or size-constrained. Ψ is anticipated to enable more efficient and streamlined solutions in achieving these goals.

### 3.2 Infering depth via minimal sensing

In this section, we explore the functionality of our TinyDepth and TinyDepth-S (the smaller variant) models, testing them on out-of-domain samples and in real-world robotics experiments. We compare our models’ performance against industrial and research-grade benchmarks such as the Intel Realsense D435i and Intel MiDaS (Ranftl et al., 2022). We significantly save power, weight, size, and cost while maintaining competitive performance. We test our depth estimation method 2.2 on two hardware platforms–one aerial and one ground. The aerial bee drone weighs 278 g and measures 92×92×84 mm, whereas the tiny car weighs only 128 g and measures 70×58×32 mm.

**FIGURE 7:**
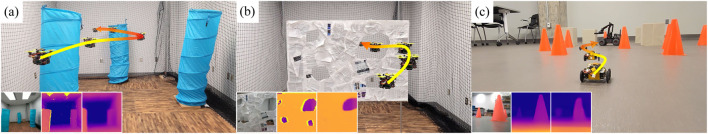
Real-world robot experiments included: **(A)** Drone navigation in an unstructured indoor forest scene, **(B)** flying through unknown gaps, and **(C)** navigating a tiny car through an obstacle course. In the lower-left corner of each image section, insets display the input RGB image, ground truth data, and depth prediction, arranged from left to right. *Note: A gradient line from yellow to red indicates the robots’ path over time, with red denoting a later temporal stage.*

Our autonomous vehicle operates in an unstructured and unknown environment as depicted in Figure 7C. This experiment aims to navigate towards a specified goal direction represented by the vector $v_{g}$ , which acts as a global guidance vector. Additionally, a local vector $v_{l}$ is determined by segmenting the depth map into depth-based binary classifications of *safe* and *unsafe* regions. We focus solely on the top half of the image, as the bottom half is typically obscured by the ground or floor. The vector $v_{l}$ is defined as the geometric center of the segmented mask’s largest *safe* region.

Combining the global and local vectors, the desired direction $v_{d}$ is calculated as a weighted sum of the two as expressed in Equation 7:

$$v_{d}=\gamma v_{l}+\left(1-\gamma \right)v_{g}$$ (7)

This approach is designed to allow vl to influence vd more significantly when obstacles are nearby. The weight γ is determined by the proximity of the nearest obstacle Zmin in the depth map, formulated as Equation 8:

$$\gamma =\frac{1}{1+e^{-\frac{1}{Z\text{min}}}};\gamma \in \left[0,1\right]$$ (8)

A Proportional-Integral-Derivative (PID) controller navigates this complex scene, ensuring precise and adaptive movement control. The car follows the control policy until it successfully reaches its destination. We showcase the effectiveness of our autonomous driving control policy in an unfamiliar obstacle course, achieving a success rate of 90% in navigating the course without any collisions across 50 trials.

For the drone experiments, we follow similar policies but in three dimensions, along with an attention mechanism. In this setup, the drone is equipped with our TinyDepth sensor suite in all four directions. It activates its obstacle avoidance policy in the direction closest to the drone.

### 3.3 Navigating with coded apertures

Integrating predicted metric depth from our CodedVO (Shah et al., 2024b) model, which utilizes a coded aperture, into existing RGB-D VO frameworks has demonstrated notable improvements in odometry. We assessed our odometry results using the standard indoor ICL-NUIM dataset (13) and our own UMD-CodedVO dataset, which includes challenging scenes like DiningRoom and Corridor characterized by low texture surfaces. These testing sequences were excluded from our training dataset to maintain an unbiased evaluation. As our foundational odometry framework, we selected ORB-SLAM2 with the loop closure feature disabled. The Absolute Trajectory Error (ATE) is the odometry accuracy evaluation metric.

**FIGURE 8:**
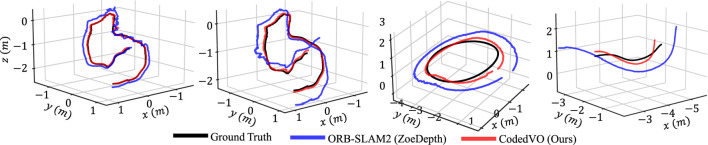
Trajectory comparison is conducted (from left to right) on the ICL-NUIM dataset (13) (specifically the of-krt2 and lr-krt2 sequences) and the UMD-CodedVO dataset (Dining and Corridor sequences). We evaluate the performance of ORBSLAM2 when using both ZoeDepth and our Coded Depth (L) as depth inputs for the ORB-SLAM2 algorithm.

We compare our approach against existing methods, categorized into those using (a) traditional RGB sensors and (b) RGB-D sensors, such as the Intel D435i. For evaluations involving traditional RGB sensors, ATE is computed after scale recovery, except in the case of ORBSLAM2-Zoe. ORBSLAM2-Zoe utilizes an RGB-D variant of ORBSLAM2 (Mur-Artal and Tardós, 2017) that incorporates RGB and metric depth from ZoeDepth (Bhat et al., 2023) as inputs. Figure 8 compares visual odometry trajectories derived from a monocular RGB sensor. It is important to highlight that our qualitative evaluation focuses exclusively on ORBSLAM2-Zoe, as the top-performing method provides camera trajectories with a known scale. By utilizing the L loss and incorporating optical constraints from phase mask coded optics, we attain state-of-the-art monocular visual odometry with a known scale, significantly reducing the size, area, weight, and power requirements of typical of robotic systems. We achieved a notable average Absolute Trajectory Error (ATE) of 0.08 m on the standard ICL-NUIM odometry dataset.

We showcase the efficacy of optical constraints using a 1-inch monocular RGB sensor equipped with a coded aperture for visual odometry. This research is intended to be a foundational step in harnessing optical and defocus constraints, opening up new possibilities for compact and resource-limited robotic systems. More details can be found here (Shah et al., 2024b) https://prg.cs.umd.edu/CodedVO.

### 3.4 Microsaccades-inspired neuromorphic camera

In this experiment, we outline the design of our AMI-EV system (section. 2.3.2 and highlight its benefits, particularly its ability to maintain stable and high-quality informational output. To showcase the system’s potential in advancing robotics perception research, we conducted evaluations using various leading-edge event-based algorithms across multiple standard applications. The findings confirm that our proposed system significantly enhances performance in all tested scenarios. For more experimental details, please refer to (He et al., 2024).

**FIGURE 9:**
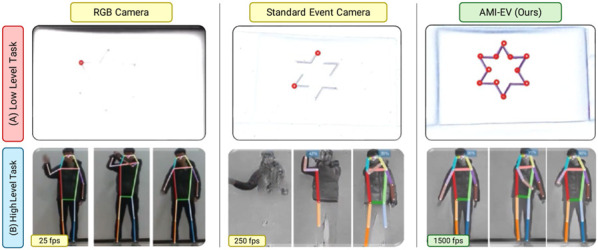
Comparsion of our system with RGB cameras and standard event cameras in both low-level and high-level vision tasks. We chose corner detection and tracking as the low-level task and high-framerate human detection and pose estimation as the high-level task.

We tested our method on low-level and high-level tasks (see Figure 9). For low-level tasks, we perform corner detection and tracking in high-luminosity conditions. The upper row of Figure 9 shows that corner detection fails in the case of RGB cameras due to weak feature corners in such adverse conditions. The texture stability in the standard event camera (S-EV) was compromised due to varying motion, leading to incomplete corner detection and inconsistent tracking. Despite this, our system and S-EV demonstrated superior performance to standard cameras under challenging lighting conditions, benefiting from the event sensor’s high dynamic range. Furthermore, our system maintained tracking for a significantly longer duration than S-EV. This accuracy discrepancy primarily stems from numerical errors and imperfect clock synchronization during AMI compensation, making it independent of camera movement.

For high-level tasks, we perform human pose estimation. For standard RGB cameras, images are restricted to 25fps due to large motion blur in RGB images that eventually leads to failure in human pose estimation. In S-EV, we see low-quality reconstruction using events even at 250 fps. This is due to bad texture stability in the S-EV. Our AMI-EV method outperforms standard RGB and event cameras in framerate and human pose estimation accuracy. We achieve significantly better accuracy even at 1500 fps.

We have developed and tested a texture-enhancing event vision system that emulates the biological microsaccade mechanism, enabling high-quality data association. Our system, which includes a rotating wedge filter placed before an event camera, successfully maintains a stable texture appearance and delivers high informational output. A comprehensive development can be found in this technical report (He et al., 2024).

## 4 Discussion

Mobile robots are becoming vital in many industries, enhancing safety and efficiency and allowing for new uses in agriculture and disaster response. Perception is at the heart of these robots’ success—the ability to understand and interact with their surroundings. This includes collecting and processing data to navigate and identify objects, enabling robots to work independently in various environments, including dangerous or remote ones. This capability makes robots more adaptable and useful in more complex situations. To achieve scalability, sustainability, and distributability in these robots, small palm-sized robots must be built capable of performing tasks like pollination or inspecting tight spaces like bridges or thermonuclear plants. Due to their size limitations, these small robots face challenges due to limited computing power and sensor capabilities. Inspired by living beings like honeybees, jumping spiders, and hummingbirds, we have developed a Minimal Perception framework at the heart of building the next-generation of palm-sized autonomous robots.

Balancing efficiency and efficacy is crucial and often task-specific. The goal is to optimize hardware and software setups to acquire essential information while minimizing redundant data. For instance, a coded aperture may cause images to appear blurry and perform poorly in texture-less environments. However, for tasks such as general navigation in texture-rich environments, the coded aperture can be both efficient and effective because depth estimation is essential. At the same time, sharp images are redundant, and the lack of texture is not an issue. Conversely, for tasks requiring indoor navigation through long corridors combined with object recognition, the advantages of a coded aperture may diminish. In such cases, to maintain a balance between efficiency and efficacy, incorporating an additional RGB or ToF sensor might be the best approach. Furthermore, utilizing only uncertainty in optical flow hinders drone navigation performance, where the metric information is the key. Without metric information, the drone cannot depict if it has successfully traversed through the unknown shaped gaps (Sanket et al., 2018; Sanket et al., 2023). All in all, it becomes a task-specific user choice to design a robot for various applications. We hope that this article serves as a blueprint for designing such robots.

This framework aims to simplify and streamline the design and functionality of tiny robots, allowing them to operate autonomously within severe size and resource limitations. Emphasizing a task-centric and selective approach to perception, this framework seeks to maximize efficiency and functionality by adopting strategies observed in nature. This enabled autonomous behaviors in palm-sized robots as small as 70 mm - showcasing navigation abilities, estimation depth in all four directions, and avoidance of dynamic obstacles in the surroundings. This approach promises to enhance tiny autonomous robots’ capabilities and supports broader applications in sustainable practices, like robotic pollination, to address ecological challenges and contribute to global food security. Minimal perception is the key to building intelligent palm-sized robots and giving us a new perspective in robot autonomy for resource-constrained robotics.

## Data Availability

The original contributions presented in the study are included in the article/supplementary material, further inquiries can be directed to the corresponding author.
